# Veterinary Drug Residues in the Food Chain as an Emerging Public Health Threat: Sources, Analytical Methods, Health Impacts, and Preventive Measures

**DOI:** 10.3390/foods13111629

**Published:** 2024-05-23

**Authors:** Hazim O. Khalifa, Lamek Shikoray, Mohamed-Yousif Ibrahim Mohamed, Ihab Habib, Tetsuya Matsumoto

**Affiliations:** 1 Department of Veterinary Medicine, College of Agriculture and Veterinary Medicine, United Arab Emirates University, Al Ain P.O. Box 1555, United Arab Emirates; 201850223@uaeu.ac.ae (L.S.); mohamed-yousif-i@uaeu.ac.ae (M.-Y.I.M.); i.habib@uaeu.ac.ae (I.H.); 2 Department of Pharmacology, Faculty of Veterinary Medicine, Kafrelsheikh University, Kafrelsheikh 3351, Egypt; 3 ASPIRE Research Institute for Food Security in the Drylands (ARIFSID), United Arab Emirates University, Al Ain P.O. Box 1555, United Arab Emirates; 4 Department of Infectious Diseases, Graduate School of Medicine, International University of Health and Welfare, Narita 286-0048, Japan

**Keywords:** veterinary drugs, drug residues, food-animal products, human exposure, residues, analytical techniques, immunological methods, preventive measures, cancer, antimicrobial resistance, public health hazards

## Abstract

Veterinary medications are necessary for both contemporary animal husbandry and food production, but their residues can linger in foods obtained from animals and pose a dangerous human risk. In this review, we aim to highlight the sources, occurrence, human exposure pathways, and human health effects of drug residues in food-animal products. Following the usage of veterinary medications, pharmacologically active compounds known as drug residues can be found in food, the environment, or animals. They can cause major health concerns to people, including antibiotic resistance development, the development of cancer, teratogenic effects, hypersensitivity, and disruption of normal intestinal flora. Drug residues in animal products can originate from variety of sources, including water or food contamination, extra-label drug use, and ignoring drug withdrawal periods. This review also examines how humans can be exposed to drug residues through drinking water, food, air, and dust, and discusses various analytical techniques for identifying these residues in food. Furthermore, we suggest some potential solutions to prevent or reduce drug residues in animal products and human exposure pathways, such as implementing withdrawal periods, monitoring programs, education campaigns, and new technologies that are crucial for safeguarding public health. This review underscores the urgency of addressing veterinary drug residues as a significant and emerging public health threat, calling for collaborative efforts from researchers, policymakers, and industry stakeholders to develop sustainable solutions that ensure the safety of the global food supply chain.

## 1. Introduction

Veterinary medications are pharmaceuticals that are used to manage or treat nutritional deficiencies in animals, treat or prevent various diseases in animals, and restore, repair, or modify their physical, mental, and biological processes. They play an important role in ensuring the health and welfare of animals in a wide range of settings [1]. The usage of veterinary drugs in animals is a common practice. For instance, veterinary antibiotics are extensively used in livestock and poultry farming worldwide, with projected consumption increasing from approximately 63,151 tons in 2010 to 105,596 tons by 2030 [2]. According to the European Food Safety Authority, drug residues are defined as drug residues refer to pharmacologically active substances, excipients, or their degradation products and metabolites that persist in animals, food, or the environment [3]. Drug residues may be detected in animal-derived food products like meat, eggs, and milk when inadequate veterinary practices are employed. This can occur for various reasons, including the unauthorized use of drugs and failure to adhere to prescribed drug withdrawal periods to ensure that residues do not exceed permissible limits [4]. In the United States, regulatory tolerances for approved veterinary drugs are set by the FDA under Title 21 Part 556 Tolerances for Residues of New Animal Drugs in Food [5]. Similarly, in the European Union (EU), maximum residue limits of pharmacologically active substances in animal-derived food products are tightly regulated under Commission Regulation (EU) No. 37/2010 [6].

Studies have shown that residues of veterinary drugs, antibiotics, and other pharmaceuticals in animal-derived food products can lead to adverse health effects, including antibiotic resistance, allergic reactions, and hormonal disruptions [7,8,9]. Additionally, drug residues can accumulate in the environment, affecting ecosystems and potentially entering the food chain [10,11]. A comprehensive review would synthesize current knowledge on the sources of drug residues, such as veterinary drug use and environmental contamination, while also exploring the health impacts and preventive measures needed to mitigate these risks. This review would inform policymakers, health professionals, and consumers about the importance of regulatory interventions, good agricultural practices, and monitoring programs to ensure food safety and public health protection in the face of this emerging threat. By highlighting the gaps in the research and proposing future directions, our review would contribute to advancing our understanding and management of drug residues as a critical public health issue.

## 2. Sources of Drug Residues in Farm Animals

Drug residues in farm animals can originate from various sources, posing risks to human health and food safety. Environmental contamination is a significant contributor, with studies highlighting how pharmaceuticals and their metabolites can persist in water sources, soil, and feed due to agricultural runoff and improper disposal of unused medications [11,12]. Additionally, failure to adhere to drug withdrawal periods is a critical factor. Withdrawal periods are established to ensure that drug residues in animal products fall below safe levels before entering the food supply. Neglecting these periods can lead to the presence of residual drugs exceeding the acceptable limits in meat, milk, or eggs, thereby compromising food safety [13]. Another key source is the off-label use of medications in animals, where drugs are administered for purposes or at doses not approved by regulatory authorities. This practice can result in unexpected residue levels in edible tissues due to improper dosing or an incomplete understanding of drug pharmacokinetics [14]. Addressing these sources requires integrated approaches involving regulatory oversight, education of farmers and veterinarians, and sustainable agricultural practices to minimize environmental contamination and ensure proper medication use in farm animals. Here, we will explore thoroughly the primary sources of drug residues in farm animals.

### 2.1. Environmental Contamination

**Figure 1:**
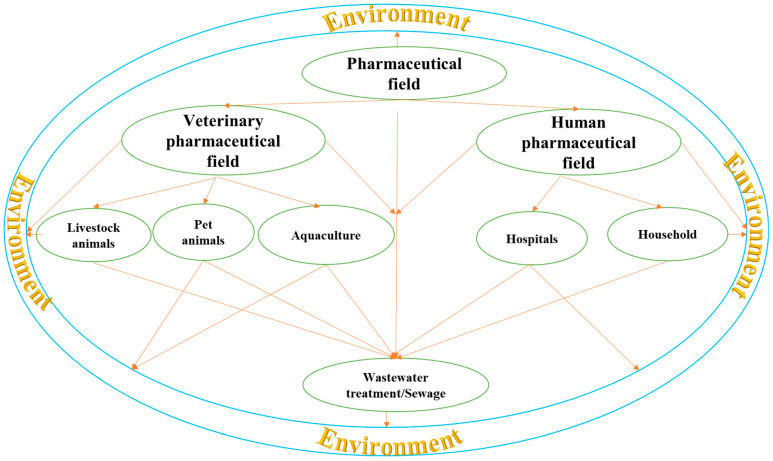
Major pathways of release of human and veterinary pharmaceuticals into the environment.

Animals can be exposed to drug residues through various means. These contaminants enter the environment from sources like farm runoffs, sewage treatment plants, and animal manure (as shown in Figure 1). Inadequate hygiene during animal transportation or product handling, inadvertent drug mixing with animal feed, and transfers from animals to their surroundings via feed or water sources are among the means by which animals may receive drug residues [12]. Livestock farmers frequently administer medications to enhance productivity and profitability. However, if these treatments are not efficiently absorbed in the intestines, the parent drug or its metabolites may be excreted in urine or feces, leading to environmental contamination [11,12,15]. Recent studies have confirmed the frequent detection of tetracyclines and their transformation products in manure and organic fertilizer samples, with concentrations ranging from 130 to 118,137 μg·kg^−1^ for tetracyclines and 54.6 to 104,891 μg·kg^−1^ for transformation products [16]. Additionally, toxicological assessments in soil environments have indicated that tetracyclines and certain transformation products could pose moderate to high ecological risks to terrestrial organisms. Furthermore, substantial amounts of drug residues resulting from direct or indirect contamination can be detected in animal manure, which is commonly utilized as fertilizer in agriculture [17]. These residues can build up in crop tissues, and eventually find their way into the food chains that feed animals and humans [17]. A recent study in China quantified 21 antibiotic and 8 non-antibiotic compounds in the collected manure-based organic fertilizers (MOFs) [2]. The researchers confirmed that antibiotics, specifically tetracyclines and fluoroquinolones, were the predominant pharmaceutical residues found in MOFs. Moreover, the authors established that antibiotics, particularly enrofloxacin and oxytetracycline present in the MOF samples, posed moderate risks to soil microorganisms and invertebrates and high risks to plants. A further possible source of drug residues could be the dust from dried liquid manure particles, which are frequently present in animal confinement facilities. Pigs, for instance, do not metabolize tetracyclines and sulfonamides efficiently and, as a result, considerable amounts of the parent medications are excreted, where they might accumulate as residues in liquid manure [18]. These medications—particularly tetracyclines—have shown to be stable and to build in environmental samples as dried liquid manure particles, which makes them a direct source of drug residue through contamination [11,19]. A meta-analysis by Frey et al. (2022) has highlighted the potential environmental contamination caused by the use of antibiotics in animal husbandry [20]. According to the study, the antibiotics used in animal husbandry have the potential to accumulate in plants and pollute nearby habitats, such as soil and water bodies. Antibiotics that are applied to agricultural land lose some of their stability. On the other hand, it was discovered that soil treated with tainted animal manure, which is applied as fertilizer, might have up to ten times the amount of veterinary antibiotics in it than is predicted to be present in the EU (PECsoil = 100 μg/kg) [21].

### 2.2. Not following a Drug’s Withdrawal Period

**Figure 2:**
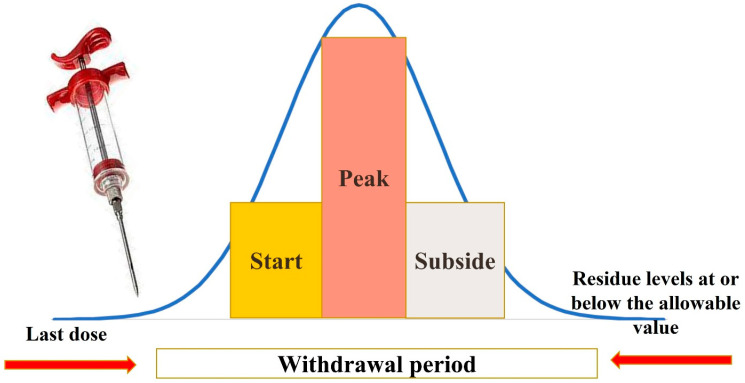
A typical illustration of a plot of the log plasma concentration curve over time, which can be obtained from plasma measurements following oral drug delivery or a single IV infusion. The withdrawal period is determined as the period of time between the last veterinary medication administration and the animal’s safe slaughter for food or safe consumption of its milk, and determined when the tolerance level for the residue concentration is at or below the allowable value.

The interval known as the withdrawal period refers to the duration between the administration of medication to an animal and the time when its milk, eggs, or meat can be sold and consumed without the risk of drug residue presence (Figure 2) [22]. During this time, the levels of drug residues in the animal’s system decrease to or below the acceptable limits (maximum residue level or tolerance level). FDA/CVM determines withdrawal periods in consumable, target tissues during the drug approval procedure. The major drug excretory organs, such as the liver and kidneys, are the most often targeted tissues. The target organ is identified, and animals are sampled at different intervals after the treatment is withdrawn during withdrawal trials. When nearly every animal administered the medication would be below the drug tolerance concentration in the target organ is determined using statistical techniques [4]. Antibiotics in food animals can leave residues in meat if withdrawal periods are not followed [13]. Studies in Africa have found high levels of drug residues in common foods [13,23]. This likely stems from antibiotic overuse, ignoring withdrawal times, and skipping veterinary advice [23,24]. Regulations on animal antibiotic use are lacking, and sick animals are sometimes slaughtered too soon for safe consumption without considering the withdrawal period [25]. For this reason, when the withdrawal period is skipped, residue ends up in animal-based commodities, like milk and meat, that are meant for human consumption [26]. It is critical to remember that a number of variables, including the type of medication used, the dosage given, and the particular animal species being treated, may have an impact on how long the withdrawal period is. Furthermore, the length of the withdrawal phase may also be influenced by the animal’s metabolism, the method, and the frequency of the medication administration (Table 1) [27,28,29,30,31,32,33]. For example, ivermectin is a drug commonly administered to food-producing animals to prevent and treat parasite infections. Ivermectin is a lipophilic drug, meaning that it remains in the animal’s edible parts for an extended period, particularly in tissues with high fat content. This characteristic of ivermectin leads to a longer withdrawal period, as well as the potential for drug residue presence in animal products derived from treated animals [34]. Additionally, this drug withdrawal period is different according to animal species and route of administration (Table 1). Therefore, ensuring compliance with the withdrawal period in different animal species is crucial for guaranteeing the safety of food products obtained from treated animals [22].

### 2.3. Extra-Label Drug Use

The practice of administering a medication in a way that deviates from the label’s instructions is known as extra-label drug use (ELDU). This can involve using the drug in ways that are not prescribed by the manufacturer, such as (a) using human drugs off-label in veterinary medicine, (b) administering drugs to non-target species, (c) treating conditions that are not approved by regulatory agencies, (d) exceeding the recommended doses, (e) administering the medication via alternative routes, (f) increasing the frequency of administration, and (g) incorrect injection techniques, and others, as reported in reference [15]. For instance, McEwen et al. conducted a study to describe the pattern of depletion of antibiotic residues (microbial inhibitory substances) in the milk of cows treated under field conditions of clinical disease and antibiotic administration, including both label and extra-label use [35]. The findings showed that, when the antibiotics are taken as directed by the label, the presence of antibiotic residues in milk beyond the withholding period is often rare. Just 3 out of 70 cases (4.3%) undergoing treatment as directed by the label had such occurrences, according to the study. The study also discovered that extra-label drug use was linked to the majority (10/13, 76.9%) of cases of extended excretion of antibiotic residues in milk. In some of these cases, particularly following the use of high doses of intramuscular or subcutaneous penicillin, milk samples remained positive until the end of sample collection. The study revealed that antibiotic residues were more likely to be observed in milk after withholding times when treatments were given in an extra-label fashion. This finding is not surprising, since withholding times for products are based on the conditions of treatment indicated on product labels and deviation from these conditions could result in a prolonged clearance of drugs from animals which, in turn, leads to a delayed elimination from milk [35]. A recent study that evaluated the oral and topical extra-label administration of fipronil to laying hens provided support for the previous findings [14]. The results were startling, in that the authors found fipronil marker residues 74 days after topical administration, and because of the drug’s extremely long withdrawal period, they did not advise the extra-label use of fipronil in laying hens under any circumstances [14]. Despite the previous fact, ELDU is still essential in the veterinary field. For instance, a recent German study confirmed that ELDU is necessary for 68.8% of cats suffering from ocular diseases, from which 96.8% received human-approved pharmaceuticals [36]. Another recent report stated that The Canadian Global Food Animal Residue Avoidance Databank receives over 2300 annual requests from licensed veterinarians, with ~65% requesting the use of extra-label drug combinations in poultry feed [37]. As a result, this review urges the responsible use of ELDU in the veterinary sector, the necessity of thorough research on the subject, and the establishment of guidelines for its usage.

## 3. Human Exposure to Drug Residues

Human exposure to antibiotic residues can occur through water, the consumption of animal products like meat, milk, and eggs, and air and dust (Table 2).

### 3.1. Drinking Water

Drinking water is one of the sources of drug residue exposure to human beings [10]. One of the primary sources of pharmaceuticals in water sources is the excretion of drugs by humans and animals and the disposal of unused medications (Table 2 and Figure 1) [38]. Pharmaceuticals can also enter water sources through industrial effluents and runoff from agricultural activities. Once these pharmaceuticals enter water sources, they can persist and accumulate in the environment, leading to the presence of drug residues in drinking water [39]. The extent of contamination can vary depending on factors such as the type and quantity of pharmaceuticals used in a particular area, the efficiency of wastewater treatment plants, and the hydrological characteristics of the water source [38]. In a study conducted in China, tap water was found to contain 17 drug residues, and found that one or more compounds were detected in 89% of the samples [40]. The drug resides included: 11 antibiotics (macrolides, sulfonamides, thiamphenicol, nitroimidazoles, trimethoprim), two nonsteroidal anti-inflammatory drugs (salicylic acid, diclofenac), a β-blocker (metoprolol), a lipid regulator (clofibric acid), a psychoactive stimulant (caffeine), and an anticonvulsant (carbamazepine).

### 3.2. Food

**Figure 3:**
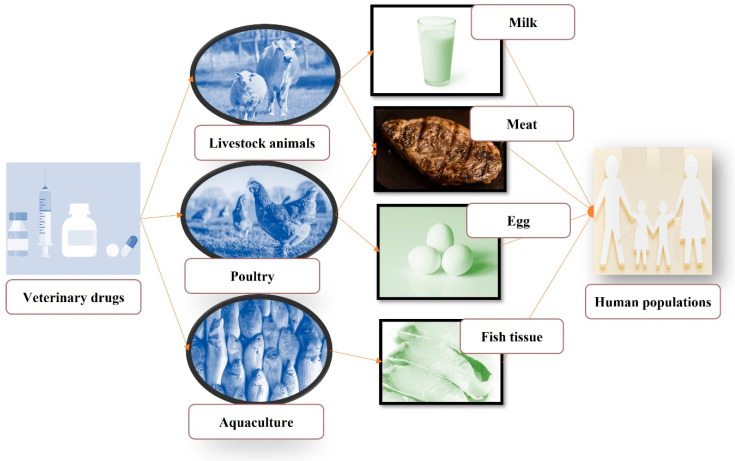
Source of veterinary drug residues in the human food chain.

Human beings are exposed to drug residues via food when residues of drugs used to treat the food animals remain in the animal’s tissues or in the food products derived from them (Table 2 and Figure 3) [4]. Drug residues can be found in meat due to the use of antibiotics, hormones, and other drugs in livestock farming, and are transferred to humans through the consumption of meat products. Antibiotic residues are the most common drug residues found in meat, with neomycin, streptomycin, penicillin, oxytetracycline, gentamicin, and sulfamethazine being the most frequently detected antibiotics [41]. Additionally, anthelmintics are another drug residues that can be found in different food products, including meat, milk, and eggs [42]. Another drug residue of concern in meat is from illegal growth hormones. Growth hormones, such as estradiol and testosterone, are used to increase the growth rate of food animals, which can lead to these residues entering the food supply and ultimately being consumed by humans [43]. In a similar manner, other food sources like milk, eggs, fish, fruit, vegetables, and honey can be the sources of drug residues in human diets [4]. Coccidiostats are potent compounds that can lead to the accumulation of residues in animal-derived food products, posing significant health risks to consumers [8]. Animals have been found to metabolize anticoccidials quickly, resulting in adverse effects. For instance, the metabolism of dimetridazole produces the main metabolite, 2-hydroxydimetridazole, which accumulates significantly in tissues and eggs [44]. The presence of anticoccidial residues in edible animal products is widespread. In Northern Ireland, detectable concentrations of lasalocid ranged from 1.5–19 µg/kg [45]. Similarly, in the UK, eggs were tested for various anticoccidials, and were found to contain residues such as salinomycin (8 µg/kg), monensin (10 µg/kg), robenidine (16 µg/kg), and lasalocid (129 µg/kg) [44]. Another crucial class of drugs in veterinary medicine is nonsteroidal anti-inflammatory drugs (NSAIDs), which are extensively utilized either alone or in combination with antibiotics to treat inflammation, pain, respiratory diseases, fever, and musculoskeletal disorders [8]. NSAIDs are also commonly employed for managing mastitis in milking cows and pigs, with residues subsequently excreted through milk [46]. Consequently, only a limited number of NSAIDs are approved for use in dairy cows. Within the EU, maximum residue limits (MRLs) have been established for various NSAIDs in milk: flunixin (40 µg/kg), meloxicam (15 µg/kg), tolfenamic acid (50 µg/kg), metamizole (50 µg/kg), and diclofenac (0.1 µg/kg) [8]. However, it has been demonstrated that certain of these residues, including diclofenac (0.13 µg/kg), tolfenamic acid (54 µg/kg), and meloxicam (15.2 µg/kg), surpass the maximum limit [46]. It is worth mentioning that the MRLs vary depending on the country, type of drug residue, and the tissue in which the residues are detected [47]. For example, the European Union has set an MRL of 100 μg/kg for sulfonamides in food of animal origin such as meat, milk, and eggs [48]. The Codex Alimentarius Commission has established MRLs of 20 μg/kg for sulfonamides in cattle milk and 100 μg/kg in animal products (muscle, fat, kidney, and liver) [49]. Another example is the MRLs for β-lactams. For benzylpenicillin and ampicillin in milk, the MRL is 4 µg/kg (4 ppb), and it ranges from 30 to 40 ppb for other antibiotics in the same group [50].

### 3.3. Air and Dust

Airborne exposure can also occur, as drugs applied to crops, animal farming, and wastewater treatment plants can volatilize and become inhalable (Table 2) [12]. This is especially an occupational hazard to workers who work with livestock, poultry, and swine, because manure from these animals contains drug residues that can volatilize and become inhalable [12,18]. Inhaled airborne drug residues enter the respiratory system and are absorbed into the bloodstream, leading to several respiratory diseases. This can occur in a variety of settings, including pharmaceutical manufacturing facilities, hospitals, and animal farms (Figure 1). For instance, a study aimed to analyze sedimentation dusts from intensive-livestock-farming barns for fluoroquinolones and estimate the association between resistant *E. coli* and the detected drugs [51]. This investigation verified that the use of drugs causes farmers and animals to swallow or inhale fluoroquinolones, as well as microorganisms resistant to these drugs. A previous study examined dust samples collected over two decades from the same piggery to assess the presence of different antibiotics [18]. The researchers confirmed that 90% of these samples contained up to five different antibiotics, including tylosin, various tetracyclines, sulfamethazine, and chloramphenicol, with total concentrations reaching up to 12.5 mg/kg of dust. Moreover, McEachran et al. (2015) found that the concentrations of three tetracyclines, tylosin and monensin, in dust found downwind of feed yards in the Southern High Plains, USA, ranged from 0.5 to 4.6 mg/kg [52]. Penicillin was found at a mean concentration of 6.6 mg/m^3^ in airborne dust from a pharmaceutical company [53]. It is important to note that antibiotics and other medications associated with dust from public spaces are not well-documented, and require thorough investigation in future studies.

**Table 2:** Different routes for human exposure to drug residues.

Method of Exposure	Description	Example	Reference
Drinking Water	Pharmaceuticals enter water sources through excretion, disposal, industrial effluents, and agricultural runoff. Once in water sources, they can persist and accumulate.	Tap water in China was found to contain 17 drug residues, including antibiotics, NSAIDs, β-blocker, lipid regulator, psychoactive stimulant, and anticonvulsant.	[38,39,40]
Food	Drug residues in food arise from the treatment of food animals with antibiotics, hormones, and other drugs. These residues can persist in meat, milk, eggs, fish, fruits, vegetables, and honey.	Meat: neomycin, streptomycin, penicillin, etc.	[41,42,43,44,45,46]
		Eggs: salinomycin, monensin, robenidine, lasalocid. Milk: NSAIDs like flunixin, meloxicam, tolfenamic acid, metamizole, diclofenac.	
Air and dust	Drug residues can volatilize from animal farming and wastewater treatment, posing an occupational hazard. Inhalation of these residues can lead to respiratory diseases.	Dust from livestock barns containing fluoroquinolones, tetracyclines, tylosin, sulfamethazine, and chloramphenicol. Penicillin in airborne dust from a pharmaceutical company.	[12,18,51,52,53]

## 4. Analytical Methods for Veterinary Drug Residues

Detection of veterinary drug residues in animal-derived foods is essential for ensuring food safety and compliance with regulatory standards. Multiclass analytical methods, which can simultaneously detect various veterinary drugs, are rapidly evolving. These methods fall into three main categories: microbiological [54,55,56], immunological [57,58,59,60,61,62,63,64,65,66,67,68,69,70,71,72,73,74], and physicochemical [75,76,77,78,79,80,81,82,83,84,85,86,87,88,89,90,91,92,93] (Table 3).

### 4.1. Microbiological Methods

Microbiological methods are typically employed as initial screening tools to detect veterinary drug residues qualitatively or semi-quantitatively. This method includes microbial inhibition test and radioactive receptor assay [54,55,56]. The microbial inhibition test detects drug residues based on their ability to inhibit microbial growth. For instance, an *Escherichia coli* strain has been used to screen for fluoroquinolone and quinolone residues in animal-derived foods by measuring the optical density changes [54,55]. The radioactive receptor assay involves the competitive binding of residues and isotope-labeled antibiotics to microbial receptors. An example of this assay is the Charm-II system, which was successfully applied to screen tetracycline and sulfonamide residues in various food products, with confirmation achieved via liquid chromatography methods [56].

### 4.2. Immunological Methods

Immunological methods leverage the specific binding properties of antibodies to detect drug residues. These methods include different assays such as enzyme-linked immunosorbent assay (ELISA), colloidal cold immunoassay (CGIA), fluorescence polarization immunoassay (FPIA), time-resolved fluoroimmunoassay (TR-FIA), biosensor technology, and quantum dots (QDs) [57,58,59,60,61,62,63,64,65,66,67,68,69,70,71,72,73,74].

#### 4.2.1. ELISA

ELISA is widely used for residue screening, due to its specificity and sensitivity. Variants of ELISA, such as heterologous and monoclonal antibody-based ELISAs, have been developed to detect residues like tetracyclines, avermectins, and fluoroquinolones in milk and other animal tissues [57,58,59]. Despite its time-consuming nature, ELISA is favored for its low cost and convenience in residue screening.

#### 4.2.2. CGIA

CGIA is often used alongside ELISA for rapid, on-site screening. For example, CGIA and ELISA have been used together to detect kanamycin and tobramycin in swine tissues, streptomycin residues in milk and pig urine, and for the detection of 19 nortesterone in pork and beef [60,61,62]. CGIA offers quick results, though it typically has a higher limit of detection compared to ELISA.

#### 4.2.3. FPIA

FPIA measures residue concentration based on the polarization of emitted fluorescence. This method has been used for sensitive, quantitative detection of gentamicin in milk, one-step FPIA for quinolone and fluoroquinolone in milk and chicken muscle and multiplexed detection of fluoroquinolones and sulfonamides in milk [63,64,65].

#### 4.2.4. TR-FIA

TR-FIA employs lanthanide chelates to enhance sensitivity and reduce background interference. It has been used to screen narasin and salinomycin residues in poultry and eggs, for the detection of chloramphenicol in shrimp and chicken muscle, and for the determination of ampicillin in cow milk [66,67,68].

#### 4.2.5. Biosensor Technology

Biosensors convert biological reactions into measurable signals. Various biosensors, including luminescent bacterial, optical, and electrochemical sensors, have been developed to detect residues like tetracyclines in poultry muscle using luminescent bacterial biosensors, amphenicol antibiotic residue detections in bovine, ovine, and porcine kidney by optical biosensors, and amperometric affinity penicillin-binding protein magnetosensors for β-lactam antibiotics in milk [69,70,71].

#### 4.2.6. QDs

QDs are semiconductor particles used in fluorescence-based assays. They have been applied to detect sulfamethazine, tetracyclines, and chloramphenicol in various animal tissues, offering high sensitivity and specificity [72,73,74].

### 4.3. Physicochemical Methods

Physicochemical methods provide high specificity and sensitivity for detecting veterinary drug residues. These methods include different assays such as liquid chromatography (LC), gas chromatography (GC), capillary electrophoresis (CE), capillary electrophoresis-mass spectrometry (CE-MS), gas chromatography–mass spectrometry (GC-MS), liquid chromatography–tandem mass spectrometry (LC-MS) [75,76,77,78,79,80,81,82,83,84,85,86,87,88,89,90,91,92,93].

#### 4.3.1. LC

LCs, including high-performance (HPLC) and ultra-high-performance (UHPLC), are widely used. These techniques separate compounds based on their distribution between stationary and mobile phases. HPLC and UHPLC with various detectors (e.g., fluorescence, diode array, UV) have been employed to detect residues such as enrofloxacin in chicken muscle, benzimidazole in farm fish, and sulfonamides in milk and meat [75,76,77,78].

#### 4.3.2. GC

GC often requires derivatization to analyze volatile derivatives. It is less frequently used, but effective for detecting compounds like chloramphenicol, lincomycin and spectinomycin, and other pharmaceuticals in animal tissues [79,80,81].

#### 4.3.3. CE

CE separates analytes based on their charge and size under an electric field. It is used for detecting sulfonamides and aminoglycosides in milk and meat, offering rapid and efficient analysis [82,83,84].

#### 4.3.4. CE-MS

CE-MS is an effective method for separating and detecting veterinary drug residues. This combination has been used successfully to determine anthelmintic benzimidazoles and their metabolites in egg samples, as well as for screening and confirming sulfonamide residues in milk [85,86]. Additionally, CE-MS techniques have been developed to detect various residues, including quinolones in bovine milk [87]. Another approach, capillary electrophoresis–quadrupole–time-of-flight mass spectrometry (CE-Q-TOF-MS), was proposed for detecting antibiotic residues in milk [88].

#### 4.3.5. GC-MS

This method has been applied to simultaneously determine various pharmaceuticals, including hormones, β-blockers, and antibacterials, at trace levels in edible animal tissues. GC-MS has also been used to detect hormones in milk, and to identify residues like 19-nortestosterone in animal tissues, with derivatization techniques enhancing the analysis [89,90,91].

#### 4.3.6. LC-MS

LC-MS combines the separation ability of chromatography with the selectivity and sensitivity of mass spectrometry. LC-MS techniques can detect a wide range of residues, such as coccidiostats, benzimidazoles, macrolides, sulfonamides, tetracyclines, penicillins, quinolones, fluoroquinolones, aminoglycosides, glucocorticoids, and steroid hormones [92,93]. This method allows for comprehensive screening and analysis of residues in various animal-derived foods, and is a standard approach in national food safety standards.

**Table 3:** Analytical methods for veterinary drug residue detection.

Category	Method	Description	Examples	References
Microbiological methods	Microbial inhibition test	Qualitative or semi-quantitative screening based on growth inhibition of microorganisms by drug residues.	Detection of fluoroquinolone residues in animal-derived foods using *E. coli* strain.	[54,55]
	Radioactive receptor assay	Competitive binding between drug residues and isotope-labeled antibiotics with a receptor on the microbial surface.	Screening tetracycline residues in food products.	[56]
Immunological methods	Enzyme-linked immunosorbent assay (ELISA)	Uses specific binding between antibodies and antigens for qualitative or quantitative analysis.	Detection of tetracycline residues in milk, monoclonal antibody-based ELISA for avermectins in milk, and dual-colorimetric ELISA for fluoroquinolone and sulfonamide residues.	[57,58,59]
	Colloidal gold immunoassay (CGIA)	Rapid screening of residues, often used alongside ELISA.	Detecting kanamycin and tobramycin residues in swine tissues, screening of streptomycin residues in milk and pig urine, and the detection of 19-nortestosterone in pork and beef.	[60,61,62]
	Fluorescence polarization immunoassay (FPIA)	Homogeneous assay based on competitive binding of the target analyte and fluorescein-labeled antigen with specific antibody sites.	Quantitative determination of gentamicin in milk, one-step FPIA for quinolone and fluoroquinolone in milk and chicken muscle, and multiplexed FPIA for fluoroquinolones and sulfonamides in milk.	[63,64,65]
	Time-resolved fluoroimmunoassay (TR-FIA)	Based on fluorescent properties of lanthanide chelates. High sensitivity and low background interference due to fluorescent properties of lanthanide chelates.	Screening narasin and salinomycin residues in poultry and eggs, the detection of chloramphenicol in shrimp and chicken muscle, and the determination of ampicillin in cow milk samples.	[66,67,68]
	Biosensor technology	Converts biological concentration into measurable signals. Diverse sensors employing biorecognition elements for detection.	Detection of tetracyclines in poultry muscle using luminescent bacterial biosensor, optical biosensor for amphenicol antibiotic residues in bovine, ovine, and porcine kidney, and amperometric affinity penicillin-binding protein magnetosensor for β-lactam antibiotics in milk.	[69,70,71]
	Quantum dots (QDs)	Semiconductor particles used for fluorescence-based detection of residues.	Indirect competitive fluorescence-linked immunosorbent assay for sulfamethazine in chicken, QD-based immunoassay for tetracyclines in bovine muscle, and QD-based lateral flow immunoassay for chloramphenicol in milk.	[72,73,74]
Physicochemical methods	Liquid chromatography (LC)	High-performance (HPLC) and ultra-high performance (UHPLC) versions separate compounds based on their interaction with stationary and mobile phases.	HPLC with fluorescence detection (HPLC-FLD) for enrofloxacin in chicken muscle, UHPLC-FLD for benzimidazole residues in farm fish, HPLC with diode array detection (HPLC-DAD) for sulfonamides in milk, and HPLC with ultraviolet (HPLC-UV) for sulfonamides in pork, liver, and chicken.	[75,76,77,78]
	Gas chromatography (GC)	Requires derivatization of analytes for volatility before detection.	GC with electron capture detector (GC-ECD) for chloramphenicol residues in animal tissues, GC with nitrogen-phosphorus detection (GC-NPD) for lincomycin and spectinomycin, and gas chromatography with an electron capture detector (GC-ECD) was utilized to detect amitraz and its metabolite residues.	[79,80,81]
	Capillary electrophoresis (CE)	High voltage electric field drives separation in a capillary channel.	CE with laser-induced fluorescence (CE-LIF) for sulfonamide residues detection in liver, solid phase extraction-capillary electrophoresis (SPE-CE) method was presented for the detection of sulfonamide residues in milk, and capillary zone electrophoresis (CZE) combined with post-column derivatization and laser-induced fluorescence detection for the determination of kanamycin, amikacin and tobramycin residues in milk.	[82,83,84]
	Capillary electrophoresis-mass spectrometry (CE-MS)	Combines CE separation with MS detection for enhanced analysis.	CE-MS for benzimidazoles in egg samples, CE-MS for screening and confirmation of sulfonamide residues in milk, CE-MS for quinolones in bovine milk, and capillary electrophoresis–quadrupole–time-of-flight mass spectrometry (CE-Q-TOF-MS) for tetracyclines and quinolones in milk.	[85,86,87,88]
	Gas chromatography-mass spectrometry (GC-MS)	Combines GC separation with MS detection	GC-MS for pharmaceuticals in edible animal tissues, GC-MS for hormones in milk, and ion trap GC-MS for 19-nortestosterone residues in animal tissues.	[89,90,91]
	Liquid chromatography-mass spectrometry (LC-MS)	Combines LC separation with MS detection for high selectivity and sensitivity.	LC-MS/MS for different veterinary drugs and pesticides in milk, and LC-MS/MS for multi-residue determination in milk powder, butter, fish tissue, and eggs.	[92,93]

## 5. Public Health Impacts

The improper use of antimicrobials as well as other veterinary medications in animal farming can give rise to issues stemming from the presence of drug residues in food and animal-derived materials [94]. This can negatively impact human health in two ways: firstly, through the direct side effects of consuming animal-origin foods containing drug residues and, secondly, indirectly, by promoting the selection of antimicrobial resistance genes that may be transmitted into human pathogens [9]. Health problems in humans resulting from prolonged exposure to sub-lethal levels of these residues include allergic reactions in individuals with sensitivities, as well as the potential for toxicity and carcinogenic effects. The major public health impacts of drug residues can be summarized into five major issues, including developing antibiotic resistance, hypersensitivity, the risk of developing cancer, teratogenic effects, and the disruption of normal intestinal flora. Furthermore, other residue-specific signs may be associated with some veterinary drug residues [95].

### 5.1. Antimicrobial Resistance

**Figure 4:**
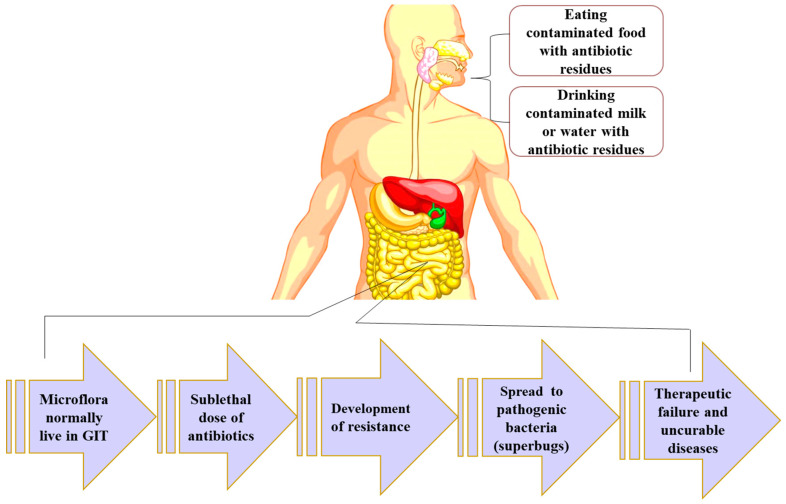
Indirect development of antimicrobial resistance due to sublethal antimicrobial residue concentrations in the gastrointestinal tract.

Antimicrobial resistance is a growing global concern in public health [96,97,98,99,100,101,102]. The rise of antibiotic-resistant bacterial infections has resulted in a significant number of deaths worldwide. A recent comprehensive analysis anticipated 4.95 million (3.62–6.57) fatalities related to bacterial AMR in 2019, with 1.27 million (95% UI 0.911–1.71) deaths attributable to bacterial AMR, and the all-age death rate due to resistance was highest in western Sub-Saharan Africa, with 27.3 fatalities per 100,000 (20.9–35.3) [103]. In both human and veterinary medicine, this issue is particularly pronounced in underdeveloped nations because of the unrestricted availability of antibiotics and inadequate infection control measures [96,97,98,99]. Shockingly, according to a global study, antimicrobial resistance claimed more lives in 2019 than either HIV/AIDS or malaria [100]. One of the key difficulties is the transmission of antibiotic-resistant microorganisms across various sectors, including humans, animals, and food chains. These microorganisms can spread to the human population through direct contact or indirect pathways, such as consuming milk, meat, and eggs. When originating in animals, these microorganisms may merge with the human body’s natural microbial community, potentially adding to the existing burden of resistance genes already found in humans [104]. The potential transfer of antibiotic resistance from animals to humans is a serious concern, given the well-established link between antibiotic use in livestock production and the emergence of antibiotic resistance in human populations. Particularly worrisome is the use of low prophylactic doses of antibiotics in animals, as it can lead to the development of antibiotic-resistant bacteria during the preparation or consumption of animal-derived foods [94]. Extensive documentation confirms that humans can acquire drug-resistant bacteria, including strains like *E. coli*, *Salmonella*, *Campylobacter*, and *Staphylococcus*, through the consumption of animal-derived foods [105,106,107]. Furthermore, certain drugs, such as fluoroquinolones and avoparcin, have been demonstrated to contribute to the emergence of antibiotic-resistant bacteria in animal-origin food sources [108]. The World Health Organization emphasizes the high priority of addressing microorganisms’ resistance arising from the subtherapeutic use of antibiotics like penicillin, tetracyclines, and sulfa drugs in agriculture [108]. Furthermore, the presence of sublethal antimicrobial concentrations in the gastrointestinal tract following the intake of contaminated food is a significant and indirect cause of the development of antimicrobial resistance (Figure 4). The normal resident bacteria in the gastrointestinal tract are more likely to develop antimicrobial resistance due to the presence of sublethal doses of antibiotics [109]. This resistance can then be easily transferred by mobile genetic elements to other pathogenic bacteria, leading to the development of superbugs which, undoubtedly, will be associated with therapeutic failures and incurable diseases. Addressing the growing threat of antibiotic resistance requires collaboration across various fields, including agriculture and veterinary medicine. This can involve replacing antibiotics in animal feed with alternative treatments to promote healthy livestock growth, a crucial component of the global food system [110]. The widespread use of antibiotics in animal feed, even for prevention as noted by Allen (2014), contaminates the environment and contributes to the development of antibiotic-resistant microorganisms [111]. Numerous researchers suggest these resistant bacteria are primarily transmitted to humans through contaminated animal products or environmentally polluted water sources [106,107,110].

### 5.2. Hypersensitivity

A drug hypersensitivity reaction refers to an abnormal immune response to a medication, typically occurring after the patient has been sensitized through previous exposure [112]. Certain drugs, including penicillin, aminoglycosides, and tetracycline, can induce hypersensitivity reactions, resulting in allergic symptoms like skin rashes, itching, hives, breathing difficulties, and even anaphylactic shock [113]. These reactions can vary in terms of severity, duration, and scope, sometimes posing life-threatening risks within minutes [114]. These effects can be triggered when individuals consume animal-derived foods containing drug residues with allergenic properties. Notably, the presence of high levels of penicillin residues in milk consumed by individuals allergic to penicillin, along with other beta-lactam antibiotics like cephalosporin, can trigger allergies [94]. Moreover, one of the main causes of drug hypersensitivity is an acute allergic hypersensitivity condition that is brought on by low concentrations of beta-lactam antibiotics in milk in a sensitized person. This is particularly concerning, as it is estimated that approximately 10% of the human population is sensitive to penicillin [112]. A drug allergy involves an immune-mediated response to a medication in a patient who has become sensitized to that drug [115]. Although drugs are foreign molecules, their molecular size is typically too small to elicit an immune response. Instead, they act as haptens, meaning they must bind to a sensitized individual’s body to become immunogenic and trigger the formation of antibodies [116]. Furthermore, penicillin-induced anaphylactic responses in beef and pork have been reported [117,118]. Meat contaminated with penicillin residues may also result in angioneurotic edema and tightness in the chest [119]. In one instance, streptomycin residues may have contributed to anaphylaxis [120]. Additionally, serum sickness, erythema multiforme, hemolytic anemia, thrombocytopenia, vasculitis, acute interstitial nephritis, Stevens–Johnson syndrome, and toxic epidermal necrolysis are the most common allergic reactions linked to beta-lactam antibiotic residues in milk [112].

### 5.3. Risk of Developing Cancer

Carcinogenic effects are associated with the impact of drugs possessing carcinogenic or cancer-inducing properties. Several countries currently use certain veterinary drugs known for their carcinogenic potential, including nitrofurans, nitromidazoles, and quinoxaline. Animal studies have demonstrated that these drug residues possess carcinogenic potential, and may pose a similar risk to humans [112,121]. These drug residues enter the human body through the consumption of animal-derived foods, carrying antimicrobial residues with the potential to be carcinogenic [112]. Another example is the residue of chloramphenicol, which raises the risk of cancer and, at very low concentrations, can cause aplastic anemia, a disorder that stops the bone marrow from producing red and white blood cells, which is frequently fatal and irreversible [122]. The inherent danger of carcinogenic residues lies in their ability to interact with or form covalent bonds with a variety of intracellular biomolecules, including proteins, ribonucleic acid (RNA), glycogen, phospholipids, and glutathione. Such interactions can lead to alterations in cellular components, particularly DNA [123]. Another concerning effect of drug residues is their potential to induce cancer by disrupting the normal biological functions of intracellular components such as DNA, RNA, proteins, glycogen, phospholipids, and glutathione through covalent bonding [4].

### 5.4. Teratogenic Effect

A pharmacological or chemical agent that can negatively affect the developing embryo or fetus, particularly during critical phases of pregnancy, is referred to as a “teratogen”. As a result, exposure to these substances while pregnant may result in congenital abnormalities that impact the organism’s structure and function. One type of antibiotic, tetracycline, is known to pass through the placental membrane and accumulate in the developing embryo’s bones and teeth. Exposure to tetracycline during pregnancy can cause yellow staining of primary (deciduous) teeth and impair the growth of long bones [124]. Prolonged consumption of certain medications or their residues at low levels can lead to teratogenic and reproductive effects. Previous reports confirmed that nitroimidazole and nitrofuran residues cause human neoplasia and the lingering effects of specific hormones [125]. The author notes that preparations can result in benign uterine abnormalities and vaginal cancer. Experimentally, oxfendazole maximum residue limit (MRL)-induced mutagenic effects in all tested cell types of tested in male and female mice and exhibited embryotoxicity, including teratogenicity in their fetuses [125]. Supporting the previous findings, Treiber and Beranek-Knauer (2021) recently summarized data from 73 scientific research that reported antimicrobial residues in commercially accessible animal products. They noted that the presence of antibiotic residues increases the frequency of mutations [126].

### 5.5. Disruption of Normal Intestinal Flora

The natural intestinal bacteria serve as a protective barrier, preventing incoming pathogens from taking hold and causing illnesses. Intestinal microorganisms serve as a crucial link between diet and human health, playing a key role in maintaining the body’s homeostasis [127]. This role is largely dependent on species diversity, the stability of the microbial community, and ecological balance. Advances in next-generation sequencing technology and bioinformatics have revealed that certain gut bacteria are closely associated with obesity [128] and cancer [129]. Recent experimental findings suggest that dysbiosis of the gut microbiome is linked to systemic inflammatory diseases, such as rheumatoid arthritis, inflammatory bowel disease (IBD), multiple sclerosis, non-alcoholic fatty liver disease, asthma, and Alzheimer’s disease [130]. Consequently, maintaining the homeostasis of the intestinal microbiota is essential. Antibiotics can decrease the overall population of these bacteria or selectively target specific essential species. Broad-spectrum antibiotics are frequently implicated in disrupting intestinal flora, leading to gastrointestinal disorders [112,121]. For instance, the use of drugs such as flunixin, streptomycin, and tylosin in animals, as well as the use of vancomycin, nitroimidazole, and metronidazole in humans, is associated with this effect [112,121]. The presence of drug residues in food products can significantly impact the ecological composition of intestinal flora. The extent of this impact depends on factors like the dosage, route of administration, bioavailability, metabolism, duration of exposure to the drug, and distribution within the body [112,121]. For example, earlier studies detecting residual amounts of antibiotics in salmon samples have been linked to alterations in normal human microflora [131]. Likewise, another study reported intestinal dysbiosis, allergic reactions, and the development of antibiotic-resistant bacterial populations following exposure to antibiotic residues in milk [132]. Furthermore, drug residues in food products can also influence the normal human microflora by reducing the population of beneficial bacteria or selectively eliminating important species. This can result in compromised intestinal barrier function against pathogenic bacteria, a condition known as intestinal dysbiosis, and an increased prevalence of drug-resistant bacteria [133]. Additionally, the human gut microbiome interacts with the antibiotic residues that are regularly consumed in drinking water. For instance, extended usage of antibiotic residues in drinking water can alter the composition of the gut microbiota and promote the growth of infectious or accidental infections that infect people [134].

### 5.6. Other Residue-Specific Signs

The classical example of residue-specific signs reported by Wu et al. (2013) on an outbreak of leanness-enhancing agent-related food poisoning that occurred in Taiwan [95]. In 2011, an outbreak of food poisoning occurred in Taiwan due to the ingestion of clenbuterol-contaminated pork, a leanness-enhancing agent. The outbreak affected 30 individuals who had consumed pork products from a single vendor. The patients exhibited symptoms such as headache, dizziness, palpitations, and sweating, which are typical of clenbuterol poisoning [95]. Clenbuterol, like other beta-adrenergic agonist drugs, was used as a partitioning agent in food animals to modify their growth [135]. It causes muscle hypertrophy and decreases fatty tissue [135]. Laboratory examination revealed that the outbreak was caused by the ingestion of pork with high levels of clenbuterol residues. The vendor responsible for the outbreak had been using clenbuterol as a growth promoter in their pigs. The authorities responded by closing down the vendor’s farm and suspending the sale of pork from the region. They also initiated a public awareness campaign to educate people about the dangers of consuming meat with drug residues. Additionally, in 2013, clenbuterol’s use as a growth agent was globally prohibited [135]. The outbreak highlighted the importance of regular monitoring and testing of food products for drug residues. Late diagnosis of such outbreaks can have severe health implications, and timely action is essential to prevent further harm.

## 6. Discussion and Potential Solutions

### 6.1. Implementing Withdrawal Periods

Earlier studies have indicated that withdrawal periods were not consistently understood or followed by various farmers [136]. Additionally, due to the expense of veterinary services, farmers frequently seek advice on antibiotic usage from non-professionals, including vendors of veterinary medications [137]. Commercially motivated drug marketers often provide antimicrobials to poultry farmers without adequate knowledge of the dosage, side effects, and withdrawal periods, exacerbating the issue [137]. The same study confirmed that approximately half of chicken farmers neglect the antibiotic withdrawal period. One of the main reasons for this is a lack of awareness among patients regarding the antibiotic withdrawal time and drug residues [137]. Additionally, a study conducted in Tanzania discovered that people were more inclined to observe withdrawal periods in certain regions [138]. In fact, many chicken farms in low- and lower-middle-income nations are run by subsistence farmers with few resources, leading to the frequent usage of antimicrobials to prevent illnesses [139,140]. Farmers face significant financial losses if they stop selling farm products (e.g., meat, milk, eggs) while using antimicrobials, therefore they continue to sell them [139,140]. Therefore, one of the most effective solutions to prevent drug residue is to regulate and enforce the use of veterinary drugs according to the directions mentioned on their label. This involves establishing clear guidelines and regulations for the use of veterinary drugs, including restrictions on the use of certain drugs (such as clenbuterol), MRLs, and withdrawal periods. The implementation of such regulations can prevent the occurrence of drug residues in food-producing animals and, ultimately, reduce the risk of AMR, carcinogenicity, toxicity, and other health effects in humans. Additionally, enforcing these regulations through regular monitoring and testing can ensure that veterinary drug use is compliant and prevent any violations [135].

### 6.2. Implement a Risk-Based Monitoring Program for Veterinary Drug Residues in Animal Food Products

This means focusing on identifying and addressing animal products that pose the greatest risks to human health. The monitoring program should include sampling, testing, and reporting of the residue levels in animal products and taking corrective actions when violations are detected. Directive 96/23/EC outlined the surveillance of veterinary medication residues in animal products until 2022. Regulation (EU) 2017/625, a more comprehensive directive that generally deals with official controls in all sectors of food production, revoked this as of 2022 [141]. According to Article 9 of this law, official controls have to be carried out appropriately often and on a risk basis, to reduce the burden on enterprises [142]. Furthermore, official regulations should also be effective across the country and across the agri-food chain. Each member state shall establish and update a multi-annual national control plan (MANCP) that details the structure and organization of official controls (EU, 2017) [141]. As a result, during the last decade, authorized food safety monitoring has shifted toward a risk-based approach [143]. The concept behind risk-based tracking is to use the majority of the resources at hand to control the existence of significant risk hazards and/or high-risk food products, while using fewer resources for low-risk hazards and/or food products, to detect more contaminated lots with the same number of resources [144,145,146]. Another study confirmed that the risk-based strategy for monitoring veterinary medicines can provide a reliable inspection priority in fishery products, which can be actively used in the construction of future national residue programs and domestic food re-inspection systems [147].

### 6.3. Educate Farmers, Veterinarians, and Consumers about the Proper Use of Veterinary Drugs and the Risks of Drug Residues in Animal Food Products

According to recent studies, antimicrobials are commonly used as growth promoters and production enhancers in layer chickens throughout many countries [148,149]. These activities violate FDA, FAO, and WHO standards for safe antibiotic use in food and agriculture [150,151,152]. Also, farmers, as well as other consumers, may consume chicken meat and eggs without understanding the risks of antibiotic residues [153,154]. Additionally, a recent Ethiopian study aimed to assess farmers’ understanding and actions regarding antibiotic usage and resistance in food animals [155]. Of the 220 cattle owners interviewed, about 80% were unable to identify what antimicrobials are and how they are utilized. Only 14.1% of respondents were aware of AMR and its effects. Another study was carried out in Kenya to investigate how antimicrobial users in the veterinary business acquire veterinary antimicrobials. The study found that the majority of antimicrobials were obtained through informal means, such as agroveterinary shops; more than half of the staff lacked the nationally mandated qualifications to advise on or sell veterinary antimicrobials, and approximately 40% of the veterinary antimicrobials were sold without a prescription [136]. Therefore, raising awareness about the benefits of prudent and responsible use of veterinary drugs, the importance of following the withdrawal period and label instructions, and the consequences of drug residues for human health are essential. It also involves providing information about alternative methods of disease prevention and control in animals such as vaccination, biosecurity, hygiene, and nutrition [8]. Furthermore, all food animals should be kept in a clean and healthy environment whenever feasible, and farmers should be encouraged to implement standard management practices and herd health programs that keep animals healthy and producing efficiently.

### 6.4. Develop New Technologies and Methods to Detect Drug Residues in Animal Products

Conventional chromatographic procedures depend on several detectors to give adequate sensitivity, specificity, and repeatability in analysis might be helpful in detecting drug residues. However, they are laborious, pricey, and ineffectual when screening a large number of samples. Veterinary drug residues in animal-derived foods can be identified utilizing modern technologies such as electrochemical biosensors [156,157], optical biosensors [158,159], MIP biosensors [160,161], and piezoelectric biosensors [162]. The sensor analysis method is simple, quick, and cost-effective, with promising findings for detecting veterinary medications in animal-derived foods. Furthermore, developing quick and portable screening tools for on-farm or on-site drug residue detection, refining analytical methods and apparatus for residue testing, and investigating cutting-edge methods to break down or remove drug residues from animal products, such as biodegradation, enzymatic hydrolysis, and nanotechnology [8]. There have been some developments in this field. One tool that can determine the concentration of antibiotics in milk samples is the Delvo incubator machine [163]. It does not need specific veterinary knowledge, and is easy to run on farms. An example of a Delvo machine is the Delvotest^®^ T from DSM-Food Specialties (Delft, The Netherlands). This broad-spectrum microbiological inhibitor test primarily detects β-lactams and tetracyclines, and it is also capable of identifying some sulfonamides, aminoglycosides, macrolides, and rifamycins in a single test. The Delvotest T kit is an enhanced version of the Delvotest SP-NT, offering improved detection capabilities for tetracyclines [164]. In the future, similar gadgets ought to be created for drug residues identification in different food sources.

## 7. Conclusions

In conclusion, the issue of veterinary drug residues represents a multifaceted challenge, with significant implications for public health. The sources of these residues are diverse, stemming from the administration of drugs to animals for therapeutic or prophylactic purposes in the agricultural industry. The health impacts on humans are a cause for concern, as exposure to veterinary drug residues through the consumption of contaminated food products can lead to adverse effects such as antibiotic resistance, allergic reactions, and potential long-term health consequences.

Preventing and mitigating the risks associated with veterinary drug residues necessitates a comprehensive and collaborative approach. This includes implementing stringent regulatory measures to monitor and control the use of veterinary drugs, enhancing surveillance systems to detect residues in food products, and promoting responsible antimicrobial use in animal husbandry. Additionally, raising awareness among stakeholders, including farmers, veterinarians, and consumers, is crucial for fostering a collective commitment to reducing the prevalence of veterinary drug residues in the food chain.

In addressing this emerging public health threat, research efforts should continue to explore alternative and sustainable practices in animal agriculture, such as promoting good farming practices, optimizing vaccination strategies, and investing in the development of innovative technologies for residue detection. By adopting a holistic and proactive stance, we can strive to safeguard public health, protect the environment, and promote a sustainable and responsible approach to veterinary drug use in the agriculture industry. Furthermore, veterinarians must take the lead in preventing drug residues by spreading knowledge among the producers, staff, and general public by being up to date on the most recent research. Additionally, the finest farm and livestock management practices, abstaining from unapproved or illegal pharmaceuticals, and using drugs appropriately, can all help to prevent drug residues.

## Figures and Tables

**Table 1:** Withdrawal periods of some important veterinary drugs in different animal species.

Animal Species	Drug	Withdrawal Period/Days	Reference
Cattle	Ampicillin	15 days (oral) and 6 days (injection)	[27]
	Chlortetracycline	10 days (oral and injection)	[27]
	Dihydrostreptomycine	10 days (oral) and 30 days (injection)	[27]
	Erythromycin	14 days (injection)	[27]
	Procaine penicillin	10 days (injection)	[27]
	Oxytetracycline	7 days (oral) and 22 days (injection)	[27]
	Sulphamezathine	7 days (oral)	[27]
	Dihydrostreptomycine	4 days (intramammary)	[28]
	Streptomycin	2 days (oral)	[28]
	Neomycin	1 days (oral) ^*^	[28]
	Ivermectin	49–66 days (subcutaneous) ^**^	[29]
Sheep and goat	Dihydrostreptomycine	30 days (injection)	[27]
	Erythromycin	3 days (injection)	[27]
	Procaine penicillin G	9 days (injection)	[27]
	Chlortetracycline	2 days (oral)	[27]
	Sulphamezathine	10 days (oral and injection)	[27]
	Sulphaquinoxaline	10 days (oral)	[27]
	Neomycin	2 days (oral)	[28]
Swine	Streptomycin	0 days (oral)	[28]
	Gentamicin	3–14 days (oral) ^***^ and 40 days (intramuscular)	[28]
	Neomycin	3 days (oral)	[28]
	Apramycin	28 days (oral)	[28]
	Ivermectin	5 days (oral)	[30]
	Levamisole	3 days (oral)	[30]
	Piperazine	21 days (oral)	[30]
	Pyrantel tartrate	1 day (oral)	[30]
	Dichlorvos	0 day (oral)	[30]
	Fenbendazole	0 day (oral)	[30]
Chickens	Streptomycin	4 days (oral)	[28]
	Gentamicin	35 days (subcutaneous)	[28]
	Chlortetracycline	1 day	[27]
	Erythromycin	2 days	[27]
	Monensin	5 days	[27]
	Tylosine	5 days	[27]
	Levamisole	0–7 days ^****^	[31]
	Ivermectin	0–12 days ^*****^ (oral)	[32]
	Nicarbazin narasin combination	5 days ^******^	[33]
	Lasalocid, salinomycin narasin, maduramicin, and semduramicin	5 days ^*******^	[33]
	Ciprofloxacin	15–19 days ^********^	[33]

^*^ The withdrawal period is reported in calves. ^**^ 49 days for products containing ivermectin as a single active substance or in combination with closantel, and 66 days when combined with clorsulon in all mammalian food-producing species. ^***^ Depending on the dose, where 3 days at a dose of 1.1 mg/kg and 14 days at a dose of 5 mg/kg. ^****^ The withdrawal times of levamisole for chicken tissue and eggs are 7 and 0 days, respectively. ^*****^ Depending on the tissue, where 12 days for liver, 8 days for skin/fat, 0 days for muscle and 10 days for kidney. ^******^ Feed withdrawal period according to the European Commission. ^*******^ Feed withdrawal period according to the Finnish National Feed Control Programme. ^********^ Feed withdrawal period according to European Health Law and National Office of Animal Health, UK.

## Data Availability

The original contributions presented in the study are included in the article, further inquiries can be directed to the corresponding author.
